# High-takeoff anomalous right coronary artery arising from the ascending aorta: a case report

**DOI:** 10.1186/s12872-026-05695-y

**Published:** 2026-04-29

**Authors:** Praveen Jeya Arul Raj, Aarthy Sundaramurthy, Harikrishnan Parthasarathy

**Affiliations:** 1 Department of Cardiology, Apollo Speciality Hospital, Vanagaram, Chennai, India; 2 Department of Radiology, Apollo Speciality Hospital, Vanagaram, Chennai, India

**Keywords:** Anomalous coronary artery, Right coronary artery, High-takeoff coronary artery, Coronary angiography, CT coronary angiography, Interarterial course

## Abstract

**Background:**

Anomalous origin of the coronary arteries is an uncommon congenital abnormality that may pose diagnostic challenges during invasive coronary angiography. High-takeoff origin of the right coronary artery (RCA) from the ascending aorta is particularly rare and can mimic ostial occlusion, potentially leading to prolonged procedures and inappropriate catheter manipulation. Computed tomography coronary angiography (CTCA) plays a crucial role in accurately defining anomalous coronary anatomy and guiding management.

**Case presentation:**

A 70-year-old woman with diabetes mellitus, hypertension, hypothyroidism, and known mitral valve prolapse presented with intermittent palpitations. Electrocardiography revealed frequent ventricular premature complexes, and 24-hour Holter monitoring documented 9,259 premature ventricular complexes. Transthoracic echocardiography showed preserved biventricular systolic function with mild-to-moderate mitral regurgitation. Invasive coronary angiography performed via the right radial artery demonstrated normal left coronary arteries but difficulty in engaging the RCA in its expected location. Following systematic angiographic exploration, the RCA was selectively engaged from a slit-like ostium located above the left coronary cusp. CT coronary angiography confirmed anomalous origin of the RCA from the anterior and left lateral wall of the ascending aorta, 20.9 mm above the sino-tubular junction, with a short interarterial course measuring 1.9 cm and no luminal narrowing or compression. The patient was managed conservatively and remained clinically stable.

**Conclusions:**

High-takeoff anomalous origin of the RCA from the ascending aorta is a rare but important diagnostic consideration when selective coronary engagement is challenging. Awareness of this entity and early use of CT coronary angiography are essential for accurate diagnosis, risk stratification, and procedural planning.

**Supplementary Information:**

The online version contains supplementary material available at 10.1186/s12872-026-05695-y.

## Background

Coronary artery anomalies are detected in approximately 0.3–1% of patients undergoing coronary angiography or cardiac computed tomography [1, 2]. With the increasing use of computed tomography coronary angiography (CTCA), particularly in intermediate- to high-risk individuals, detection rates appear higher than earlier angiographic series, reflecting improved recognition rather than a true increase in prevalence. Among coronary anomalies, anomalous origin of the right coronary artery (RCA) accounts for nearly 0.1% of cases [2]. High-takeoff RCA arising from the ascending aorta above the sino-tubular junction is exceedingly rare and has been described predominantly in isolated case reports [3]. Although many coronary anomalies are clinically silent, failure to recognize aberrant origins during invasive coronary angiography may result in diagnostic confusion, prolonged procedural time, and inappropriate catheter manipulation. CT coronary angiography provides definitive anatomical characterization and plays a key role in guiding diagnosis, risk stratification, and management planning [4–6].

## Case presentation

A 70-year-old woman presented with intermittent palpitations of several months’ duration. She denied chest pain, exertional dyspnea, syncope, or presyncope. Her medical history included diabetes mellitus, hypertension, hypothyroidism, and mitral valve prolapse. She was receiving beta-blocker therapy and amiodarone for symptomatic ventricular ectopy.

Electrocardiography demonstrated sinus rhythm with frequent ventricular premature complexes. Twenty-four-hour Holter monitoring recorded 9,259 premature ventricular complexes without sustained arrhythmia. Transthoracic echocardiography showed prolapse of the A2–A3 scallops of the mitral valve with mild-to-moderate eccentric mitral regurgitation and mild left atrial enlargement. Left and right ventricular systolic functions were preserved, left ventricular filling pressures were normal, and there were no regional wall motion abnormalities. A treadmill exercise test was positive for inducible ischemia.

Given the patient’s advanced age, multiple cardiovascular risk factors, and a positive treadmill exercise test suggestive of inducible ischemia, invasive coronary angiography was performed to evaluate for obstructive coronary artery disease despite preserved ventricular systolic function and absence of regional wall motion abnormalities.

**Fig. 1:**
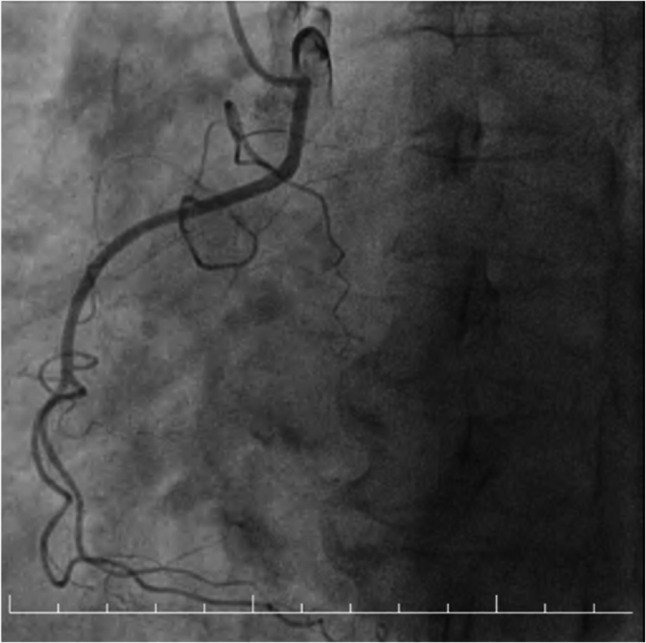
Coronary angiography demonstrating anomalous right coronary artery origin. Selective coronary angiography performed via the right radial approach showing difficulty in engaging the right coronary artery in its expected position, with subsequent visualization of the vessel arising from a high-takeoff location above the left coronary cusp

**Fig. 2:**
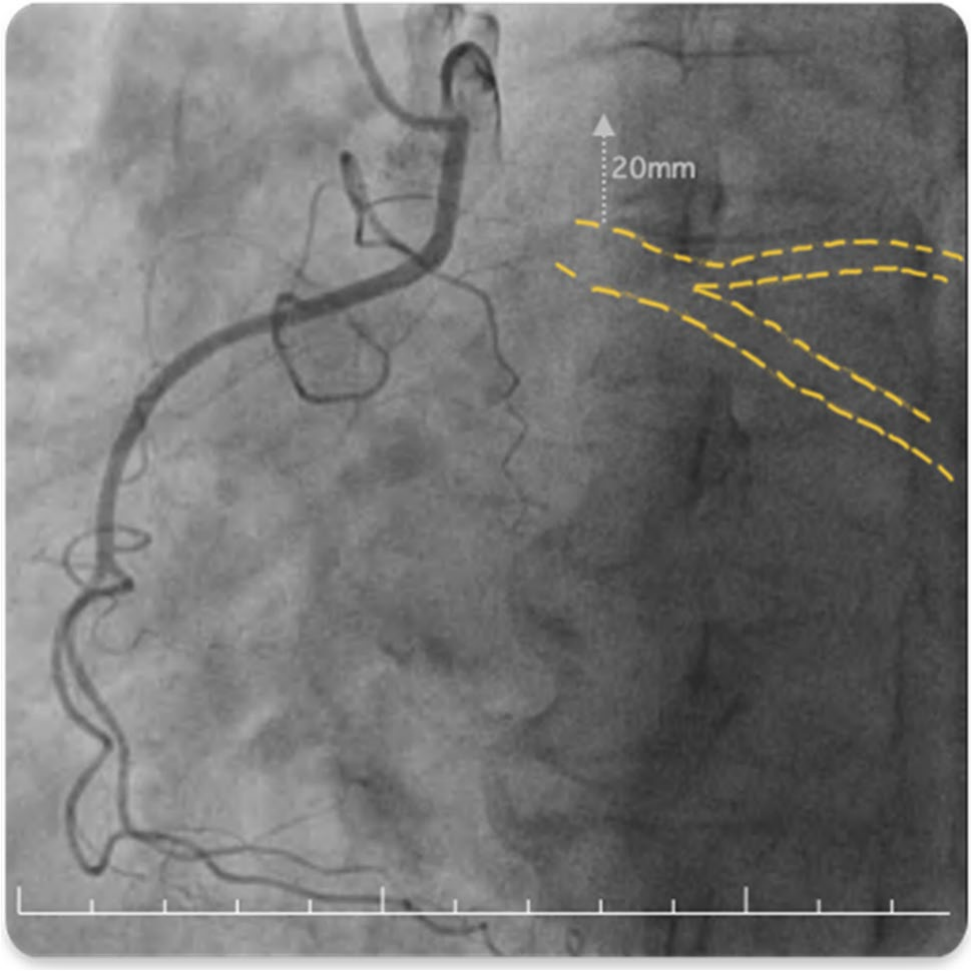
High-takeoff origin of the right coronary artery on angiography. Coronary angiographic image illustrating the slit-like ostium of the right coronary artery arising from the ascending aorta, approximately 20 mm above the sino-tubular junction (dashed lines), consistent with a high-takeoff anomalous origin

Coronary angiography was performed via the right radial artery using a 5-French sheath. The left coronary system was engaged easily with a TIG catheter and demonstrated normal epicardial coronary arteries. Multiple attempts to engage the right coronary artery (RCA) in the right coronary sinus were unsuccessful (Fig. 1). A Judkins Right catheter was subsequently used, and systematic exploration of both coronary sinuses failed to identify the RCA ostium. Careful probing of the ascending aorta using small-volume contrast injections eventually revealed a slit-like ostium located above the left coronary cusp, approximately 20 mm above the sino-tubular junction (Fig. 2). Angiography demonstrated a normal-caliber RCA without stenosis and a co-dominant coronary circulation.

**Fig. 3:**
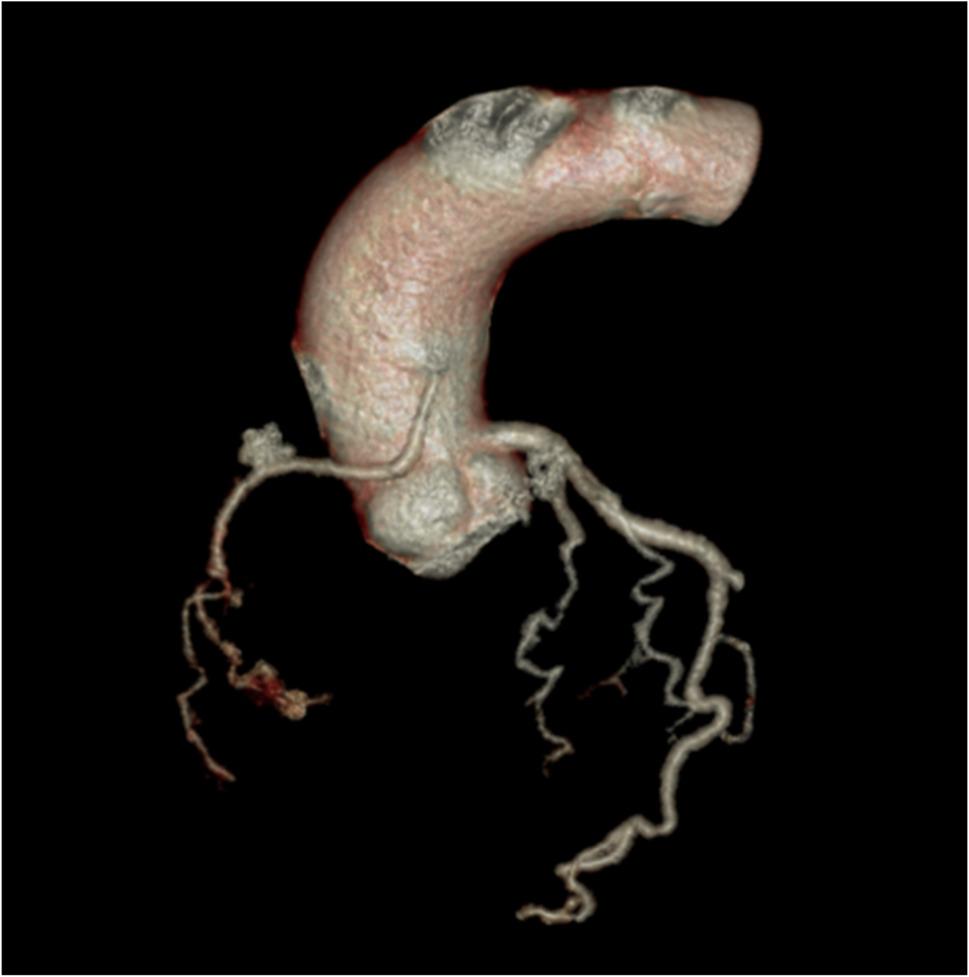
Computed tomography coronary angiography confirming anomalous origin and course. Three-dimensional volume-rendered computed tomography coronary angiography showing anomalous origin of the right coronary artery from the anterior and left lateral wall of the ascending aorta with a short interarterial course between the ascending aorta and the main pulmonary artery

Computed tomography coronary angiography confirmed anomalous origin of the RCA from the anterior and left lateral wall of the ascending aorta, 20.9 mm above the sino-tubular junction and superior to the left sinus of Valsalva. The RCA followed a short interarterial course measuring 1.9 cm between the ascending aorta and the main pulmonary artery before continuing along its usual course (Fig. 3). No luminal narrowing, intramural segment, or dynamic compression was identified.

The anomalous coronary anatomy was considered an incidental finding and was not felt to be causally related to the patient’s ventricular ectopy. The patient was managed conservatively with continuation of beta-blocker therapy and amiodarone, with reported symptomatic improvement on follow-up.

## Discussion

Coronary artery anomalies are identified in approximately 0.3–1% of angiographic and CT-based studies, with anomalous origin of the right coronary artery (RCA) reported in nearly 0.1% of cases [1, 2]. High-takeoff RCA arising from the ascending aorta represents a particularly rare variant and is most often described in isolated case reports [3]. Contemporary understanding emphasizes that the clinical relevance of such anomalies is determined primarily by proximal coronary morphology rather than the site of origin alone.

An interarterial course has historically been regarded as a high-risk feature due to its association with myocardial ischemia and sudden cardiac death. However, current consensus supports a risk continuum influenced by multiple anatomical factors, including ostial configuration, takeoff angle, presence of an intramural segment, and dynamic compression during exercise [4]. In the present case, CT coronary angiography demonstrated a short interarterial segment without luminal narrowing, intramural course, or evidence of dynamic compression, supporting a low-risk anatomical configuration. In the absence of ischemic symptoms or high-risk CT features, additional provocative ischemia testing was not pursued, and conservative management with clinical follow-up was considered appropriate.

The principal educational value of this case lies in highlighting an important diagnostic pitfall during invasive coronary angiography. Failure to selectively engage the RCA should prompt consideration of an anomalous origin rather than immediate assumption of ostial occlusion. Meticulous angiographic planning, systematic evaluation of the region above the sino-tubular junction, and thoughtful catheter selection are essential to avoid diagnostic delay and unnecessary catheter manipulation. This consideration is particularly relevant in emergency settings such as primary percutaneous coronary intervention, where delayed recognition may prolong reperfusion time. CT coronary angiography remains the gold standard for definitive anatomical delineation of anomalous coronary arteries and plays a critical role in guiding interventional or surgical planning [5, 6, 7].

## Conclusion

High-takeoff anomalous origin of the right coronary artery from the ascending aorta is a rare anatomical variant that may present a significant diagnostic challenge during invasive coronary angiography. Failure to selectively cannulate the right coronary artery should not be equated with ostial occlusion, and prompt consideration of anomalous origin is essential to avoid unnecessary catheter manipulation and procedural delay. Computed tomography coronary angiography plays a pivotal role in accurate anatomical delineation, risk stratification, and planning of interventional or surgical management.

## Supplementary Information

Supplementary Material 1.

## Data Availability

All data generated or analysed during this study are included in this published article.

## Abbreviations

RCA: Right coronary artery
CTCA: Computed tomography coronary angiography
ECG: Electrocardiogram
