# Supplementary material for: High-takeoff anomalous right coronary artery arising from the ascending aorta: a case report
Source: BMC Cardiovasc Disord. 2026 Apr 29;26:519. doi: 10.1186/s12872-026-05695-y (PMC13281287; doi:10.1186/s12872-026-05695-y)
Supplement: Supplementary file 1 — Supplementary Material 1. [file 12872_2026_5695_MOESM1_ESM.pdf]

CARE CHECKLIST

**CARE Item**	**Item Description**	**Reported in Manuscript**
**1**	Title identifies the case report and condition	Title page
**2**	Key words	Keywords
**3a**	Abstract – Background	Abstract
**3b**	Abstract – Case presentation	Abstract
**3c**	Abstract – Conclusions	Abstract
**4**	Introduction / Background explaining relevance	Background
**5a**	Patient demographics (age, sex)	Case presentation
**5b**	Main symptoms / presenting complaints	Case presentation
**5c**	Medical, family, psychosocial history	Case presentation
**5d**	Relevant past interventions and medications	Case presentation
**6**	Physical examination and clinical findings	Case presentation
**7**	Timeline of clinical events	Case presentation
**8a**	Diagnostic methods (ECG, echo, angiography, CTCA)	Case presentation
**8b**	Diagnostic challenges	Case presentation
**8c**	Diagnostic reasoning and differential diagnosis	Discussion
**9a**	Therapeutic intervention (medical management)	Case presentation
**9b**	Rationale for management decisions	Discussion
**10a**	Follow-up and clinical outcomes	Case presentation
**10b**	Follow-up test results	Case presentation
**11a**	Discussion of strengths and limitations	Discussion
**11b**	Review of relevant literature	Discussion
**11c**	Clinical implications and lessons	Discussion
**12**	Patient perspective	Not applicable
**13**	Informed consent obtained	Declarations
